# Tailoring of Self-Healable Polydimethylsiloxane Films for Mechanical Energy Harvesting

**DOI:** 10.1021/acsaem.4c01275

**Published:** 2024-09-25

**Authors:** Kalyan Ghosh, Alexander Morgan, Xabier Garcia-Casas, Sohini Kar-Narayan

**Affiliations:** † Department of Materials Science & Metallurgy, University of Cambridge, Cambridge CB3 0FS, United Kingdom; ‡ Nanotechnology on Surfaces and Plasma Group, Materials Science Institute of Seville (CSIC-University of Seville), C/Américo Vespucio 49, 41092 Seville, Spain

**Keywords:** Self-healing, PDMS, Ecoflex, Triboelectric nanogenerator, Energy harvesting

## Abstract

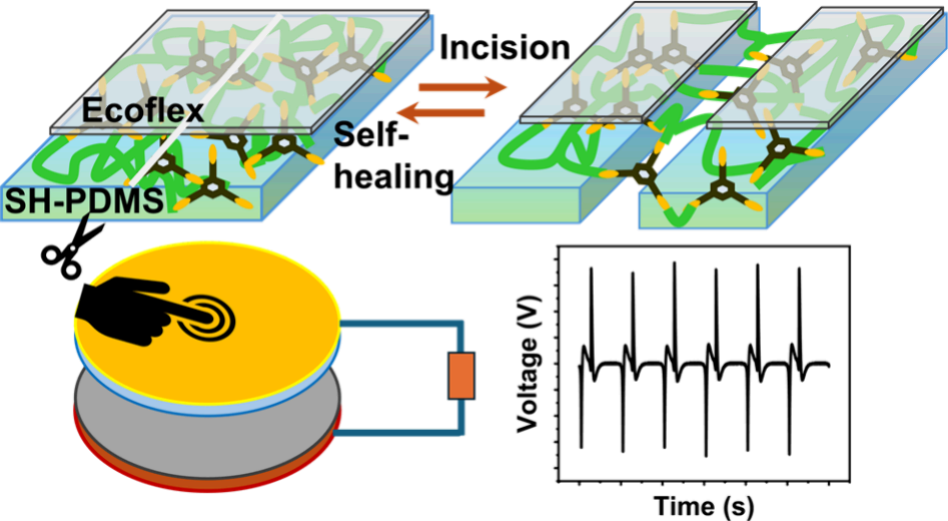

Triboelectric nanogenerators (TENGs) have emerged as potential energy sources, as they are capable of harvesting energy from low-frequency mechanical actions such as biological movements, moving parts of machines, mild wind, rain droplets, and others. However, periodic mechanical motion can have a detrimental effect on the triboelectric materials that constitute a TENG device. This study introduces a self-healable triboelectric layer consisting of an Ecoflex-coated self-healable polydimethylsiloxane (SH-PDMS) polymer that can autonomously repair mechanical injury at room temperature and regain its functionality. Different compositions of bis(3-aminopropyl)-terminated PDMS and 1,3,5-triformylbenzene were used to synthesize SH-PDMS films to determine the optimum healing time. The SH-PDMS films contain reversible imine bonds that break when the material is damaged and are subsequently restored by an autonomous healing process. However, the inherent stickiness of the SH-PDMS surface itself renders the material unsuitable for application in TENGs despite its attractive self-healing capability. We show that spin-coating a thin layer (≈32 μm) of Ecoflex on top of the SH-PDMS eliminates the stickiness issue while retaining the functionality of a triboelectric material. TENGs based on Ecoflex/SH-PDMS and nylon 6 films show excellent output and fatigue performance. Even after incisions were introduced at several locations in the Ecoflex/SH-PDMS film, the TENG spontaneously attained its original output performance after a period of 24 h of healing. This study presents a viable approach to enhancing the longevity of TENGs to harvest energy from continuous mechanical actions, paving the way for durable, self-healable mechanical energy harvesters.

## 1 Introduction

The fast advancement of technology is giving rise to a revolution in our daily lives with the development of flexible electronics, including e-textiles, sensors, transistors, the Internet of Things, and multimedia devices.^1−4^ To apply these electronics in various practical settings, the emergence of a power source that is both robust and flexible with a long cycle life is a significant challenge.^5,6^ Conventional power sources like batteries and supercapacitors are capable of meeting the energy demand; however, they require periodic charging and have limited lifespans, considerable weight, inflexibility, and often produce toxic waste.^7,8^ Therefore, there is a growing demand for energy sources that possess adaptable capabilities, are environmentally friendly, and have extended lifespans.^9−12^ In this context, mechanical energy harvesting is particularly attractive, as there is a plentiful amount of ambient mechanical energy in nature, such as wind, the movement of plant leaves, rainfall, human locomotion, vehicles in motion, and ocean waves.^13−15^

In 2012, Fan et al. introduced the concept of a triboelectric nanogenerator (TENG), which is a device capable of converting small-scale mechanical energy from the surrounding environment into electrical energy using the principles of triboelectrification and the electrostatic induction phenomenon.^16^ Several models of TENGs have been developed over the past years such as (a) vertical contact-separation mode, (b) sliding mode, (c) single-electrode mode, and (d) free-standing mode.^17,18^ Among them, a contact-separation mode TENG (CS-TENG) consists of two distinct dielectric materials and their respective current collectors. The dielectric materials are brought into contact periodically, resulting in surface charge transfer. Empirical versions of triboelectric series serve to categorize materials according to their propensity to either gain or lose electrons upon interaction with other materials.^19−21^ Materials on opposing ends of the triboelectric series have very different electron affinities, which makes them suitable candidates for TENG applications because of the improved electric charge transfer between them.^22,23^ Polydimethylsiloxane (PDMS), fluorinated ethylene propylene (FEP), polyvinylidene fluoride (PVDF), and polytetrafluoroethylene (PTFE) are commonly classified as potential negative triboelectric materials or tribonegative materials, and human skin, hair, nylon 6, and cotton wool are typically classified as potential positive triboelectric materials or tribopositive materials.^20,24−26^ TENGs typically comprise appropriate combinations of tribopositive and tribonegative materials. However, the continuous exposure of TENGs to external mechanical forces such as bending, twisting, pressing, and sliding can result in mechanical damage to the triboelectric layers and subsequent dysfunction. Therefore, it is desirable to incorporate an artificial self-healing capability into TENGs, inspired by the natural attributes of living creatures.^27^ This capability would allow for the spontaneous repair of damage and the recovery of functionality. Recently, researchers have developed several self-healable materials for energy harvesting and storage applications.^27−30^ Self-healable polymers based on shape-memory polyurethane,^31^ poly(1,4-butylene adipate) and disulfide bonds,^32^ imine or hydrogen (H)-bond linked PDMS,^33−37^ and polyvinyl alcohol/agarose hydrogel^38^ have been used as dielectric layers in TENGs. Among them, imine or H-bond linked self-healable polydimethylsiloxane (SH-PDMS) draws special attention due to its ability to spontaneously heal at ambient temperature without any additional treatment.^39^ For example, Sun et al. fabricated only imine bond-based SH-PDMS, employing aminopropyl-terminated PDMS and 1,3,5-triformylbenzene to fabricate self-healable, stretchable, transparent TENGs.^33^ Later, Cai et al. fabricated double-cross-linked PDMS by altering the ratio of imine bonds to H-bonds through a reaction of aminopropyl-terminated PDMS with isophorone diisocyanate (IPDI) and terephthalaldehyde (TPAL). Optimizing the ratio of IPDI and TPAL to the PDMS, the SH-PDMS can be healed within 2 h.^36^ In another report, Cui et al. fabricated SH-PDMS by reacting hexamethylene diisocyanate (HDI) with aminopropyl-terminated PDMS. The self-healability is established through the H-bonds that are formed between the adjacent HDI-modified PDMS linear chains through the urea motifs.^35^ Recently, Du et al. presented self-healing composites of polydimethylsiloxane–polyurea and graphite/carboxylated carbon nanotube complex fillers with multiple H-bonding cross-linking networks. The authors showed that H-bonding networks can break apart and rearrange when exposed to a small quantity of ethanol, providing fast healing in 3 h with 99.4% healing efficiency at room temperature.^37^ However, in spite of its efficient self-healing capability, SH-PDMS is intrinsically sticky and adheres to other surfaces naturally.^40−43^ This behavior severely restricts its application for vertical CS-TENG, as additional peeling force is required to separate the SH-PDMS layer from the opposite triboelectric layer. In this work, we investigate whether a thin coating of a nonadhesive layer with comparable triboelectric behavior could mitigate the stickiness of the top surface of SH-PDMS while simultaneously retaining the self-healing capability.

Here, we have identified Ecoflex, which belongs to the silicone elastomer family, as an ideal material for coating on SH-PDMS. Ecoflex is an extremely flexible and stretchable material that is compatible with living organisms, can be restored to its original shape while stretched, and is environmentally friendly when compared to other types of elastic materials.^44,45^ It also shows good triboelectric properties because of its silicon–oxygen chemical backbone (−Si–O−), flexibility, and robust mechanical properties.^46,47^ To construct a highly efficient CS-TENG device, it is imperative to consider the opposite tribopositive electric material to pair with Ecoflex, which is tribonegative. From the triboelectric series, nylon is a potential tribopositive material among synthetic polymers.^11,21,25,48−50^ Nitrogen atoms in the repeating amide units of nylon have lone pairs of electrons that can be transferred from its surface to the electronegative Ecoflex/PDMS surface, thereby generating localized positive and negative charges on their respective surfaces, leading to contact electrification.^25,48^

Hence, in this study, we fabricated Ecoflex-coated SH-PDMS (Ecoflex/SH-PDMS) films as the negative triboelectric material in a CS-TENG device, thereby mitigating the stickiness issue of SH-PDMS while simultaneously retaining its self-healing property. Ecoflex has been chosen as the coating material, as it is compatible with SH-PDMS since they both belong to the silicone family and possess a similar chemical backbone (−Si–O−). The SH-PDMS undergoes self-repair at room temperature after a certain interval of time, akin to human skin. The spontaneous healing of SH-PDMS holds the Ecoflex layer on top intact and restores its properties. We show that the Ecoflex/SH-PDMS composite film can be freely incorporated into the CS-TENG device geometry as the stickiness issue is resolved. The CS-TENG is constructed by employing nylon 6 as the tribopositive layer and the Ecoflex/SH-PDMS film as the tribonegative layer, which are brought into periodic contact through external mechanical excitation at a frequency of 2 Hz. We show that the Ecoflex/SH-PDMS|nylon 6 CS-TENG regained its energy generation ability with almost ≈99% recovery efficiency even after multiple incisions were introduced to the Ecoflex/SH-PDMS layer. This work therefore presents a robust method of tailoring the surface of the self-healable triboelectric PDMS layer to make it suitable for real-life applications as a power source for next-generation self-powered electronics that rely on the harvesting of energy from mechanical actions.

## 2 Experimental Section

### 2.1 Materials

Bis(3-aminopropyl)-terminated polydimethylsiloxane, average *M*_n_ of ∼27 000 g mol^–1^, 1,3,5-triformylbenzene (TFB), dimethylformamide (DMF), and isopropyl alcohol (IPA) were purchased from Merck, UK. Nylon 6 film (thickness of 100 μm) was procured from Goodfellow, UK. High Temp Resin for stereolithography (SLA) 3D printing of molds was purchased from Formlabs, UK. Ecoflex 00-10 was purchased from Smooth-On, UK. Conductive copper (Cu) tape for electrical connections was obtained from Teraoka, Japan.

### 2.2 Fabrication of 3D-Printed Molds

The molds used for casting the SH-PDMS films were designed by employing Autodesk Fusion 360 software, as shown in Figure S1. The designed shapes were printed by a Form 3 3D printer (Formlabs) using High Temp Resin. The 3D-printed molds were washed with IPA in Form Wash (FormLabs) and cured in a Form Cure (Formlabs) station at 80 °C for 2 h. The molds were then used for casting of SH-PDMS and Ecoflex films.

### 2.3 Fabrication of SH-PDMS Films

The SH-PDMS was fabricated by reacting bis(3-aminopropyl)-terminated polydimethylsiloxane (abbreviated as NH_2_-PDMS-NH_2_) and TFB at different molar ratios following a modified route as reported by Sun et al.^33^ At first, 0.8 M TFB was prepared in DMF. The solution was sonicated in an ultrasonic bath (Branson 3800) for 15 min to obtain a clear solution of TFB. Three different categories of SH-PDMS films were prepared, employing 500 mg of NH_2_-PDMS-NH_2_ with 25, 50, and 100 μL of 0.8 M TFB to obtain 0.05, 0.1, and 0.2 mL g^–1^ TFB/NH_2_-PDMS-NH_2_ ratios, respectively. The NH_2_-PDMS-NH_2_ and TFB mixtures were stirred vigorously for 2–5 min and immediately cast onto SLA-printed molds. A double-sided conductive Cu tape that acted as the current collector was placed in the groove first, and using the doctor blade technique, the viscous mixture was cast to the edge of the groove. The addition of Cu tape in the mold prior to casting facilitated quicker removal of the film from the mold and elimination of additional conductive coating steps later that would have been required to add the current collector. The mixtures were allowed to stand for 12 h at room temperature inside a fume cupboard and then cured at 120 °C for 7 h in an electric oven (Heratherm, Thermo Scientific). The films with attached Cu tape were carefully removed from the mold. For each category of mixtures, five samples were prepared and analyzed. The SH-PDMS films with ratios of 0.05, 0.1, and 0.2 mL g^–1^ TFB/NH_2_-PDMS-NH_2_ are denoted as SH-PDMS_0.05_, SH-PDMS_0.1_ and SH-PDMS_0.2_, respectively.

### 2.4 Fabrication of Pure Ecoflex and Ecoflex-Coated SH-PDMS Films

Similar to the SH-PDMS film preparation described above, Ecoflex film was prepared following the same casting technique, mixing Part A and Part B of Ecoflex 00-10 with a 1:1 ratio for 10 min and casting the mixture onto the 3D-printed molds. The mixture was cured at room temperature for 4 h. To fabricate the Ecoflex-coated SH-PDMS films, the SH-PDMS_0.1_ film was spin-coated using a raw mixture (≈50 mg) of Part A and Part B (1:1) of Ecoflex 00-10 at 2500 rpm for 1 min employing a spin coater (Laurell Technologies WS-650MZ-23NPPB). The Ecoflex-coated SH-PDMS_0.1_ (Ecoflex/SH-PDMS_0.1_) was kept for 4 h at room temperature for curing and subsequently used for CS-TENG fabrication. Following the same fabrication technique, varying the depth of mold and the amount of raw mixture of Part A and Part B (1:1) of Ecoflex 00-10, we varied the thickness of the SH-PDMS_0.1_ and Ecoflex layers.

### 2.5 Materials Characterization

The surfaces of all of the films were imaged with scanning electron microscopy (SEM) using a Hitachi TM303PLUS desktop microscope at a 15 kV voltage in secondary electron (SE) and backscattered electron (BSE) modes. SEM images of the cross sections of the films were recorded to determine the thickness of SH-PDMS_0.1_ and Ecoflex layers. ImageJ software was used to analyze the thickness of the individual layers, taking averages from three similar samples. The X-ray diffraction (XRD) study was carried out using a Bruker D8 Advance diffractometer in Bragg–Brentano geometry with a Cu–Kα_1,2_ source (λ = 1.5406 Å) and a LynxEye EX position sensitive detector scanning from 2θ of 5° to 80°. Fourier-transform infrared (FTIR) spectroscopy was carried out using a Nicolet iS50 FTIR spectrometer (Thermo Scientific) in attenuated total reflectance (ATR) mode. To determine the surface roughness of the films, a DektakXT stylus profilometer (Bruker) was used, running in 3D map scan mode for a 1 × 1 mm^2^ area and applying a stylus force of 1 mg for soft Ecoflex/PDMS films and 3 mg for nylon 6 film. Vision64, Bruker’s 64-bit parallel processing software, was used for data analysis. To demonstrate the self-healability of the films, several incisions 5–6 mm in length were made on the surfaces of Ecoflex/PDMS films using a razor blade and then left for healing at room temperature. The locations of the incisions were imaged using SEM after defined time intervals.

### 2.6 TENG Output Measurement

The TENG output was measured in contact-separation mode, employing Ecoflex/SH-PDMS_0.1_ as the negative triboelectric material and nylon 6 film as the positive triboelectric material. A schematic diagram of the device is shown in Figure S2. The as-purchased nylon 6 film was cut in the shape of a table tennis bat with a circle having a diameter of 12.5 mm and an attached length of 10 mm (Figure S2) using a Zing 16/24 laser cutter (EpilogLaser). Gold was sputter-coated on nylon 6 with a thickness of ≈100 nm using a Desk Sputter Coater-DSR1 sputter coater (VAC COAT). A linear motor (LinMot) was employed for periodic contact and separation of the two triboelectric layers at a frequency of 2 Hz. The average force employed between the two triboelectric layers of 3.7 N was measured using AEP transducers, type TCA load cell 10 kg. The contact area between nylon 6 and Ecoflex/SH-PDMS_0.1_ was calculated as 1.22 cm^2^. A bespoke setup was built to measure the triboelectric performance employing the Ecoflex/SH-PDMS_0.1_ and nylon 6. The nylon 6/Au film was attached to the linear motor device, and the Ecoflex/SH-PDMS_0.1_/Cu electrode was attached at the opposite side on a glass substrate connecting to the force sensor. The lower contact frequency and testing force were chosen to replicate operating conditions similar to ambient mechanical actions such as human motions. The output voltage profile (*V*) was measured using a digital oscilloscope (Tektronix TBS 2000B Series) connected to a resistor box with multiple resistances to determine the conditions for optimal impedance matching to determine the highest power output from the device. The average power output per cycle (*P*_avg_) was determined by numerically integrating the instantaneous power dissipated in the ohmic resistance (*R*) for *n* cycles of period (*T*) and dividing by the total time period (Δ*t* = *t*_1_ – *t*_0_*= nT*) using eq 1.
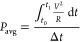
1

To perform these calculations and to control the automated acquisition of the output signal for long periods of time, a customized data processing software interface (NanoDataLyzer) built on the MATLAB app designer (R2023b) was used. The measurements were recorded after tapping for 1 min, i.e., 120 cycles, to stabilize the surface charges. A cyclic test was conducted for 16 h by capturing the voltage profile at regular intervals of 30 min, connecting to an external load of 104 MΩ and keeping all other parameters the same. Additionally, incisions 7–8 mm in length were introduced on the surface of the Ecoflex/PDMS_0.1_ film at different locations using a razor blade, and the film was left for healing for about 24 h. Following this healing step, a cyclic stability test was conducted on the TENG device for a further 7 h to determine whether the triboelectric energy harvesting performance had been restored posthealing.

## 3 Results and Discussion

**Figure 1:**
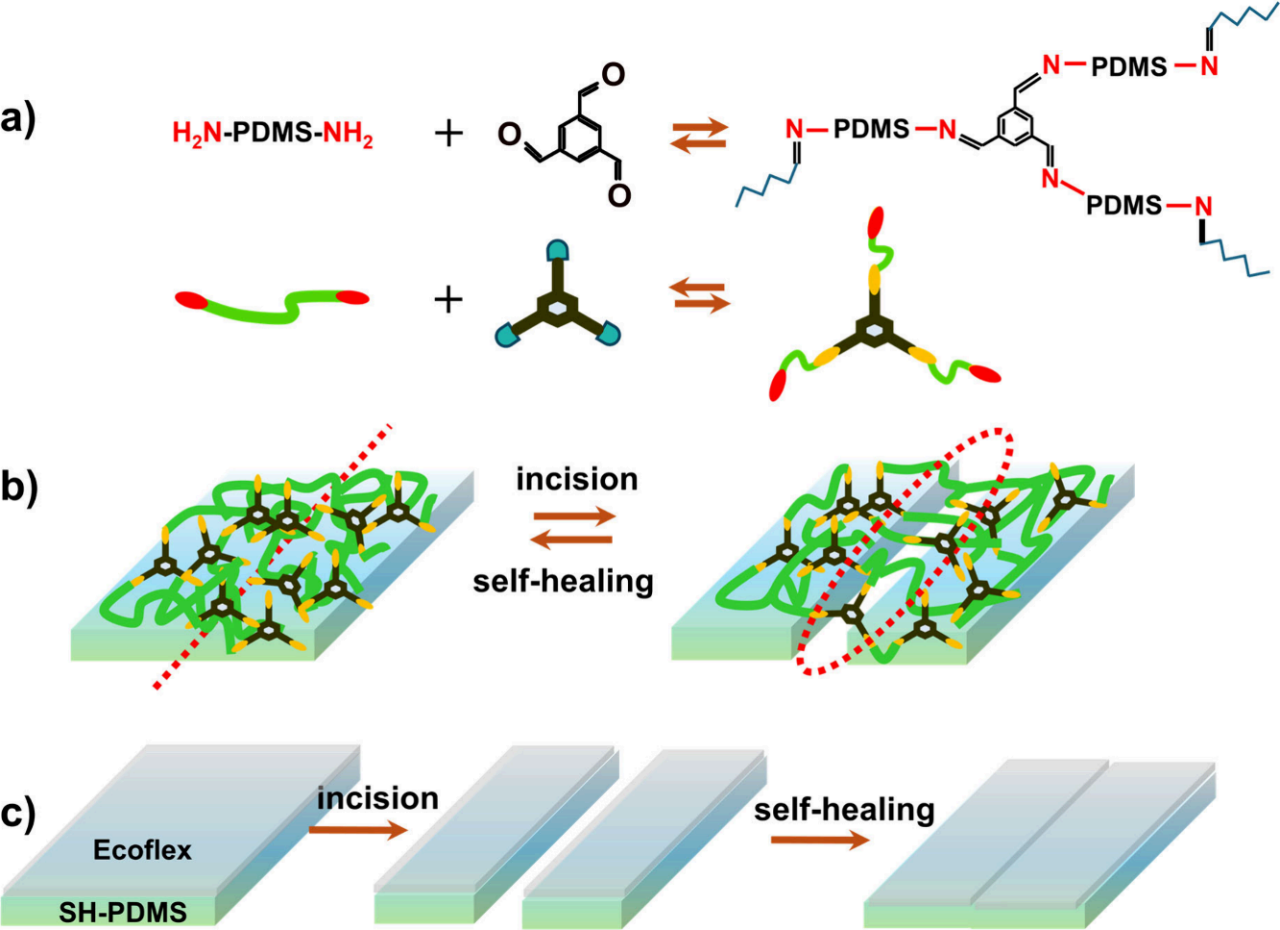
Schematic representation of the self-healing process. (a) Chemical reaction between NH_2_-PDMS-NH_2_ and TFB, showing the imine bond formation for cross-linking and healing of PDMS. Healing process of (b) SH-PDMS and (c) Ecoflex/SH-PDMS films. (a, b) Adapted from ref (33). Copyright 2018 American Chemical Society.

The CS-TENG device was fabricated by employing Ecoflex/SH-PDMS_0.1_ film as the negative triboelectric layer and nylon 6 film as the positive triboelectric layer. In this report, we have only shown the self-healing nature of the Ecoflex/SH-PDMS film. The SH-PDMS film was fabricated by first optimizing the ratio of NH_2_-PDMS-NH_2_ and 1,3,5-triformylbenzene (TFB). The amine functional groups (−NH_2_) and formaldehyde (−CHO) groups of the TFB react to form imine bonds following the Schiff base reaction.^51^ The trifunctional TFB facilitates cross-linking of the PDMS chains. The imine bond in this context serves as both a cross-linking point and a healing point. When the film is incised, the imine bonds break and are hydrolyzed, leading to the creation of initial aldehyde and amine compounds. During the healing process, the two incised surfaces come in contact for a specific duration under ambient conditions; the aldehyde and amine bonds react spontaneously to produce the imine bond. The reversible imine bond facilitates the self-healing of the PDMS film.^33,34^ The chemical reaction between NH_2_-PDMS-NH_2_ and TFB as well as the healing process of the SH-PDMS and Ecoflex/SH-PDMS films are shown in Figure 1.

**Figure 2:**
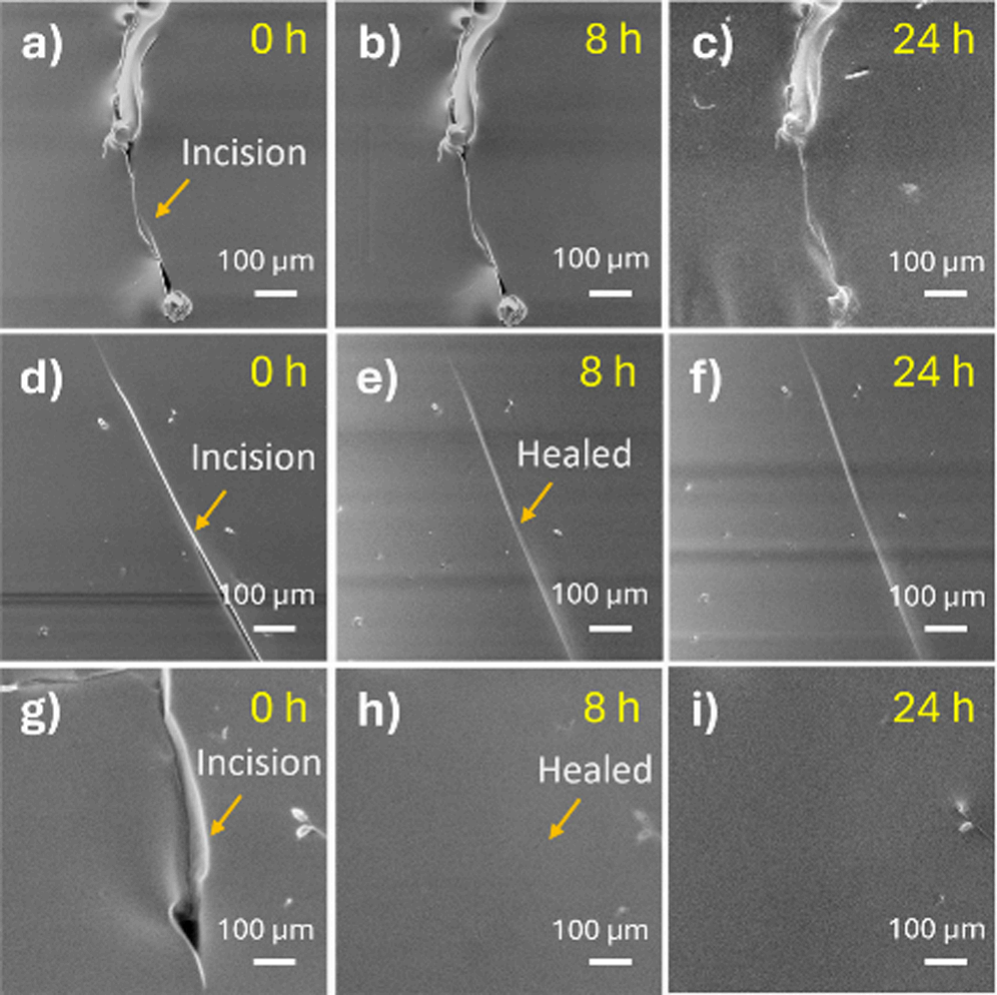
SEM images displaying the time-dependent healing process of (a–c) SH-PDMS_0.05_, (d–f) SH-PDMS_0.1_, and (g–i) SH-PDMS_0.2_.

We varied the mixing ratios of NH_2_-PDMS-NH_2_ and TFB to obtain the optimum healing time. With an increasing content of TFB, the healing time was reduced. A systematic study was carried out to determine the healing time for SH-PDMS_0.05_, SH-PDMS_0.1_, and SH-PDMS_0.2_ films for further surface modification. The SEM images of the incised films at the time intervals of 0, 8, and 24 h are shown in Figure 2. It was observed that both the SH-PDMS_0.1_ (Figure 2d–f) and SH-PDMS_0.2_ (Figure 2g–i) films were healed by 8 h. However, in the SH-PDMS_0.1_ film, the incision mark was found to persist, even after healing. The SH-PDMS_0.05_ film (Figure 2a–c) took a longer time to heal, about 24 h. Additionally, the width of the incision also played a role. For a sharp incision, when the faces of the two cut edges were closer, healing was found to be faster. A similar observation is noticed for human skin, where a deeper cut to the skin requires a suture to heal. Observing the healed images, SH-PDMS_0.2_ was found to exhibit the best healing performance. However, the synthesis of the SH-PDMS_0.2_ film was harder to control, as the mixture of NH_2_-PDMS-NH_2_ and TFB only provided a short (∼2–3 min) time interval for casting, as the reaction starts almost instantaneously. On the contrary, a longer time was available for casting during the synthesis of SH-PDMS_0.05_ and SH-PDMS_0.1_ films. Considering the shelf life of the reactant mixture and the self-healing time of all of the films, the SH-PDMS_0.1_ film was chosen for further modification to investigate its triboelectric performance in a CS-TENG device.

**Figure 3:**
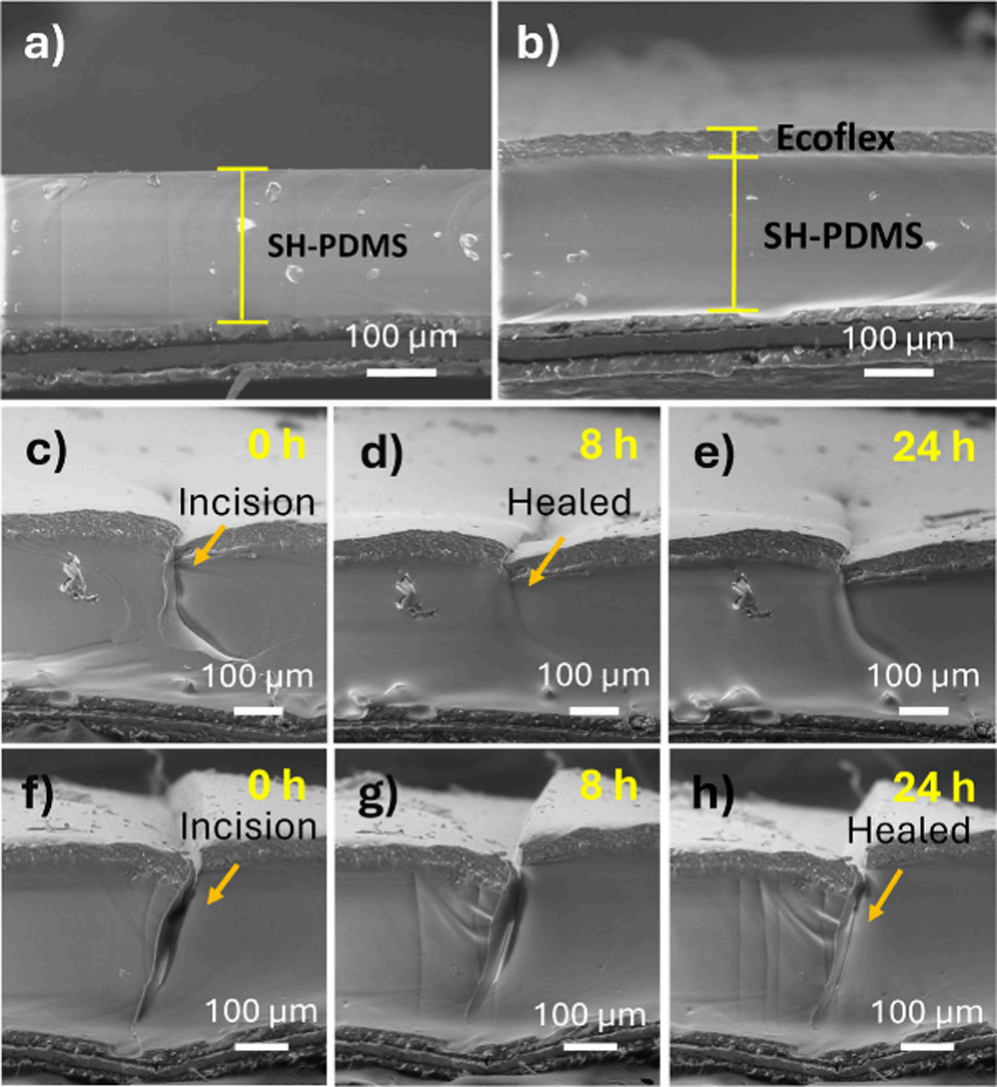
SEM images of the cross sections of (a) SH-PDMS_0.1_ and (b) Ecoflex/SH-PDMS_0.1_ films. The time-dependent healing process of Ecoflex/SH-PDMS_0.1_ for a (c–e) sharp and (f–h) broad incision.

Although the SH-PDMS_0.1_ film showed a suitable self-healing performance, the film’s surface was sticky, thereby easily adhering to other materials, making it unsuitable as a triboelectric layer. A thin coating of Ecoflex elastomer was added on top of the SH-PDMS_0.1_ layer by using a spin coater to prevent stickiness. SEM images of the cross sections of SH-PDMS_0.1_ and Ecoflex/SH-PDMS_0.1_ films are presented in Figure 3a,b. The average thickness of the SH-PDMS_0.1_ film from the three samples was measured to be 200 ± 4 μm, while the coating of the Ecoflex layer was measured to have an average thickness of 32 ± 2 μm. To study the effect of the thickness of the Ecoflex and SH-PDMS_0.1_ layers on the triboelectric performance, we fabricated additional films of Ecoflex/SH-PDMS_0.1_. The SEM images of the cross sections of all of the films are shown in Figure S3. For each category, three samples were analyzed. The average thicknesses of the individual Ecoflex/SH-PDMS_0.1_ layers of the films were measured to be 32 ± 2/300 ± 4, 32 ± 2/450 ± 4, 50 ± 2/300 ± 4, and 85 ± 2/300 ± 4 μm.

A comparison of the stickiness of the SH-PDMS_0.1_ and Ecoflex/SH-PDMS_0.1_ films against a piece of paper is shown in Video S1. It was noticed that the SH-PDMS_0.1_ film spontaneously adhered to the paper substrate and required an additional force to separate the two layers, whereas the Ecoflex/SH-PDMS_0.1_ film did not adhere to the paper substrate. The self-healability of the Ecoflex/SH-PDMS_0.1_ composite film was investigated by cutting the film at different locations with sharp and broad incisions, as shown in Figure 3c,f. The surface with a sharp incision was found to heal within 8 h (Figure 3d,e), while for the broad incision, about 24 h of healing was required (Figure 3g,h). The healing of the underlying SH-PDMS layer was found to drag the cut faces of the Ecoflex layer on top into intimate contact (Figure 3e), thus healing the Ecoflex layer as well.

**Figure 4:**
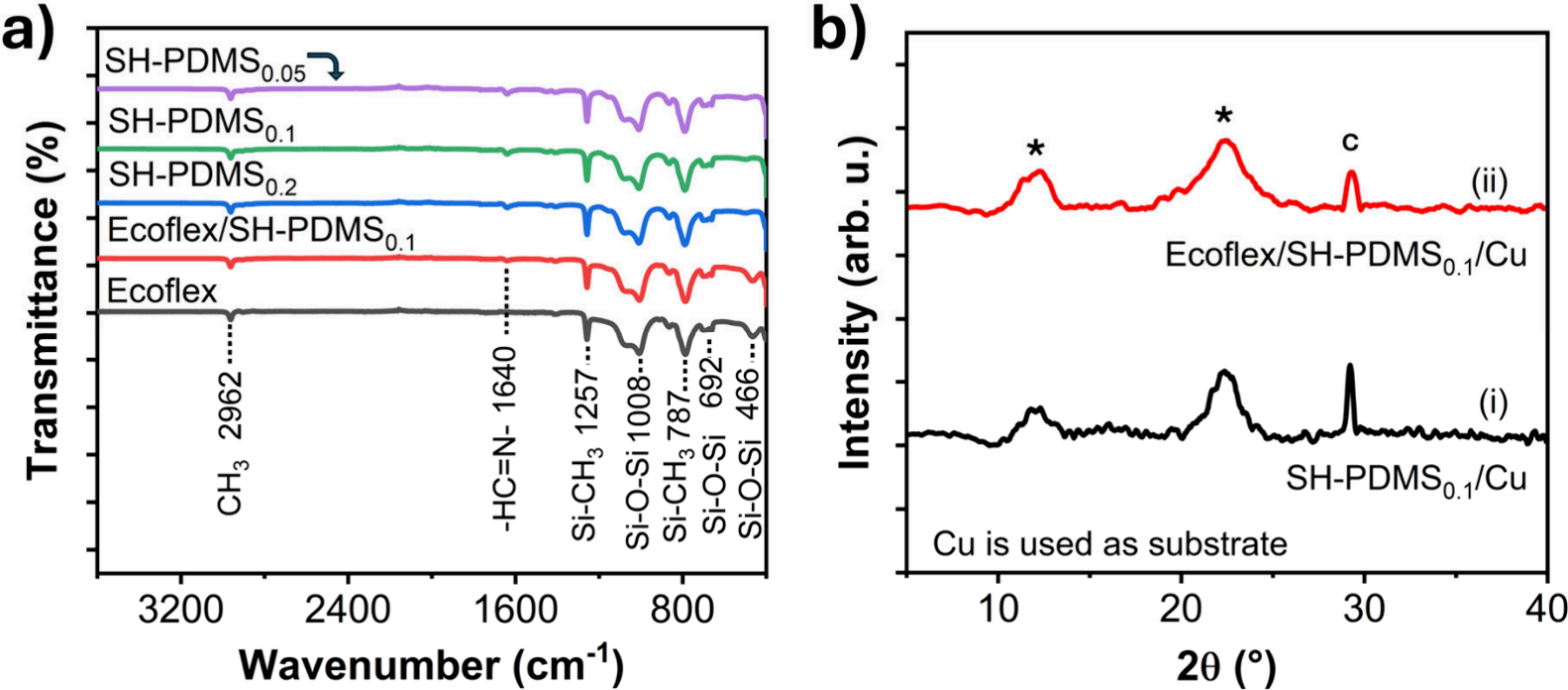
(a) FTIR spectra of SH-PDMS_0.05_, SH-PDMS_0.1_, SH-PDMS_0.2_, Ecoflex/SH-PDMS_0.1_, and blank Ecoflex films and (b) XRD patterns of SH-PDMS_0.1_ and Ecoflex/SH-PDMS_0.1_. Cu film was used as a substrate to cast the films.

FTIR spectroscopy was carried out to confirm the successful formation of SH-PDMS and Ecoflex films from the raw precursors. The FTIR spectra of SH-PDMS_0.05_, SH-PDMS_1.0_, SH-PDMS_0.2_, Ecoflex/SH-PDMS_0.1_, and blank Ecoflex films are shown in Figure 4a. All the SH-PDMS films show a transmission peak at 2962 cm^–1^, corresponding to C–H bond stretching vibrations in −CH_3_ groups. The peaks at 1257 and 787 cm^–1^ are attributed to the Si–C stretching vibrations of Si–CH_3_ and Si–(CH_3_)_2_, respectively. The peaks observed at 1008, 692, and 466 cm^–1^ correspond to the stretching of Si–O–Si bonds in the PDMS (silicone elastomer) backbone. Additionally, all the SH-PDMS films show an additional peak at 1640 cm^–1^, corresponding to the imine bond (C=N) stretching vibration that confirms the formation of the dynamic imine link by the reaction between NH_2_-PDMS-NH_2_ and TFB.^52−55^ The blank Ecoflex shows all the characteristic peaks of silicone rubber, similar to PDMS, confirming the successful formation of silicone elastomer through mixing an equivalent amount of Part A and Part B components of Ecoflex 00-10. The Ecoflex/SH-PDMS_0.1_ shows all the peaks present in Ecoflex, along with an additional peak of lower intensity at 1640 cm^–1^, corresponding to the imine bond of SH-PDMS.

The XRD patterns of SH-PDMS_0.1_ and Ecoflex/SH-PDMS_0.1_ are shown in Figure 4b. The SH-PDMS_0.1_ shows broad diffraction peaks at 2θ of 12.1° and 22.4° (marked with *), corresponding to the amorphous silicone elastomer.^56,57^ Ecoflex/SH-PDMS_0.1_ shows the same diffraction peaks corresponding to the combined effect from Ecoflex and PDMS_0.1_.^57,58^ The peak marked with “c” corresponds to the crystalline peak from the Cu film, which was included in the fabrication step as a substrate to cast the SH-PDMS films. The XRD patterns of Ecoflex/SH-PDMS_0.1_ before and after the self-healing are presented in Figure S4a. As XRD patterns show the diffraction peaks related to the crystal structure, the peak positions remained unchanged before the incision and after the self-healing. The XRD pattern of nylon 6 film is presented in Figure S4b.

A 3D map scan was carried out using a DektakXT profilometer for both Ecoflex/SH-PDMS_0.1_ and nylon 6 films. The false-color 3D plots of the surface of Ecoflex/SH-PDMS_0.1_ at four locations with a 1 × 1 mm^2^ surface area are shown in Figure S5. A variation of the height difference was noticed at different locations because of the uneven template surface. The surface roughness parameters, root-mean-square (RMS) roughness (*R*_q_) and arithmetic roughness average (*R*_a_), from four locations are presented in Table S1. The average values of *R*_q_ and *R*_a_ are calculated to be 2.425 ± 0.634 and 1.925 ± 0.556 μm, respectively. The 3D map scan of commercial nylon 6 is presented in Figure S6.

**Figure 5:**
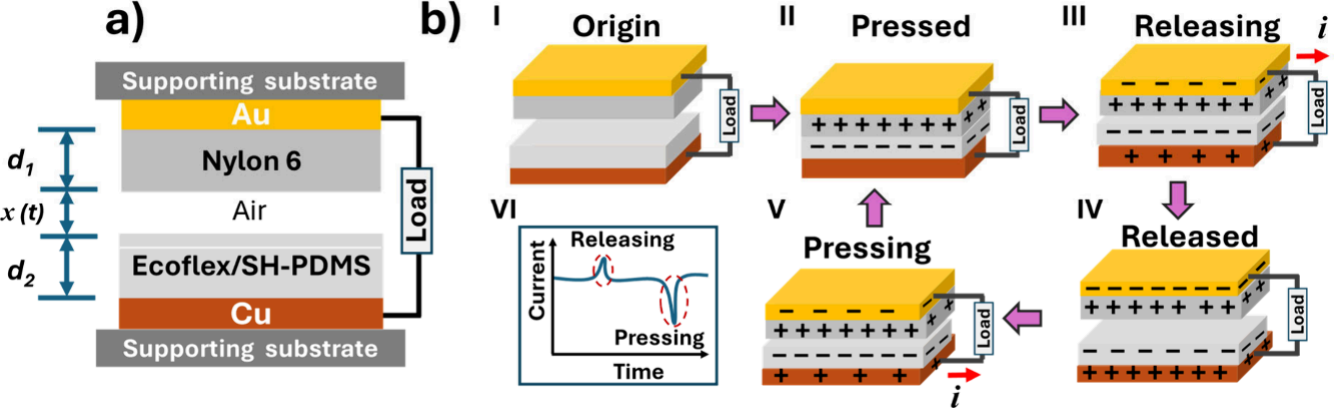
(a) Schematic of a cross-sectional view of the CS-TENG. (b) (I–V) Different charge transfer states during the contact-separation process of nylon 6 and Ecoflex/SH-PDMS and(VI) schematic of obtained alternate current response from a contact-separation cycle. Adapted from ref (59). Copyright 2012 American Chemical Society.

A schematic diagram of the cross section of the CS-TENG device is shown in Figure 5a. The device operates in contact-separation mode, where Ecoflex/SH-PDMS_0.1_ and nylon 6 are two triboelectric layers, and the Cu and Au layers act as electrodes. The operational principle of the CS-TENG device involves the combination of contact electrification and electrostatic induction during periodic contact and separation of the Ecoflex/SH-PDMS_0.1_/Cu and nylon 6/Au layers. Before the contact-separation process, all of the layers are neutral (Figure 5b-I). During the pressing step (Figure 5b-II), contact electrification happens and distributes electrostatic charges with opposite signs on the two surfaces of the polymer films.^59^ The nylon 6 film loses electrons and becomes positively charged, while the Ecoflex/SH-PDMS_0.1_ film gains electrons and becomes negatively charged. This charge distribution creates an interface dipole layer. At the releasing step (Figure 5b-III), when the nylon 6 film moves away, electrostatically induced free charges flow across the external load between the two adjacent Cu and Au electrodes.^16^ Due to this electrostatic induction effect, negative charges are induced on the attached Au electrode of nylon 6, and positive charges are induced on the attached Cu electrode of the Ecoflex/SH-PDMS_0.1_ film. This flow of electrons in the external circuit due to the electrostatic induction effect provides an output signal. When the two plates are entirely separated, the surface charge is completely neutralized, and no electric signal is detected (Figure 5b-IV). During the succeeding pressing step, the electrons reverse their flow, resulting in the emergence of a reverse electric signal (Figure 5b-V).^16^ Thus, a continuous periodic contact-separation of the two layers produces alternating current signals, as depicted in Figure 5b-VI.^59^

According to theoretical studies on TENGs, the output voltage (*V*) of a contact-separation mode dielectric-to-dielectric TENG is given by eq 2:^18,60^
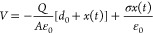
2where *A* is the surface area of the triboelectric layer, *Q* is the transferred charge, ε_0_ is the permittivity of air, σ is the generated triboelectric charge density, *x*(*t*) is the time-dependent distance between the two triboelectric layers, and *d*_0_ is the effective dielectric thickness, which is given by eq 3:
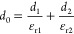
3where *d*_1_ and *d*_2_ are the thicknesses of the two dielectric materials and ε_r1_ and ε_r2_ are the relative dielectric constants of the two dielectric materials.

**Figure 6:**
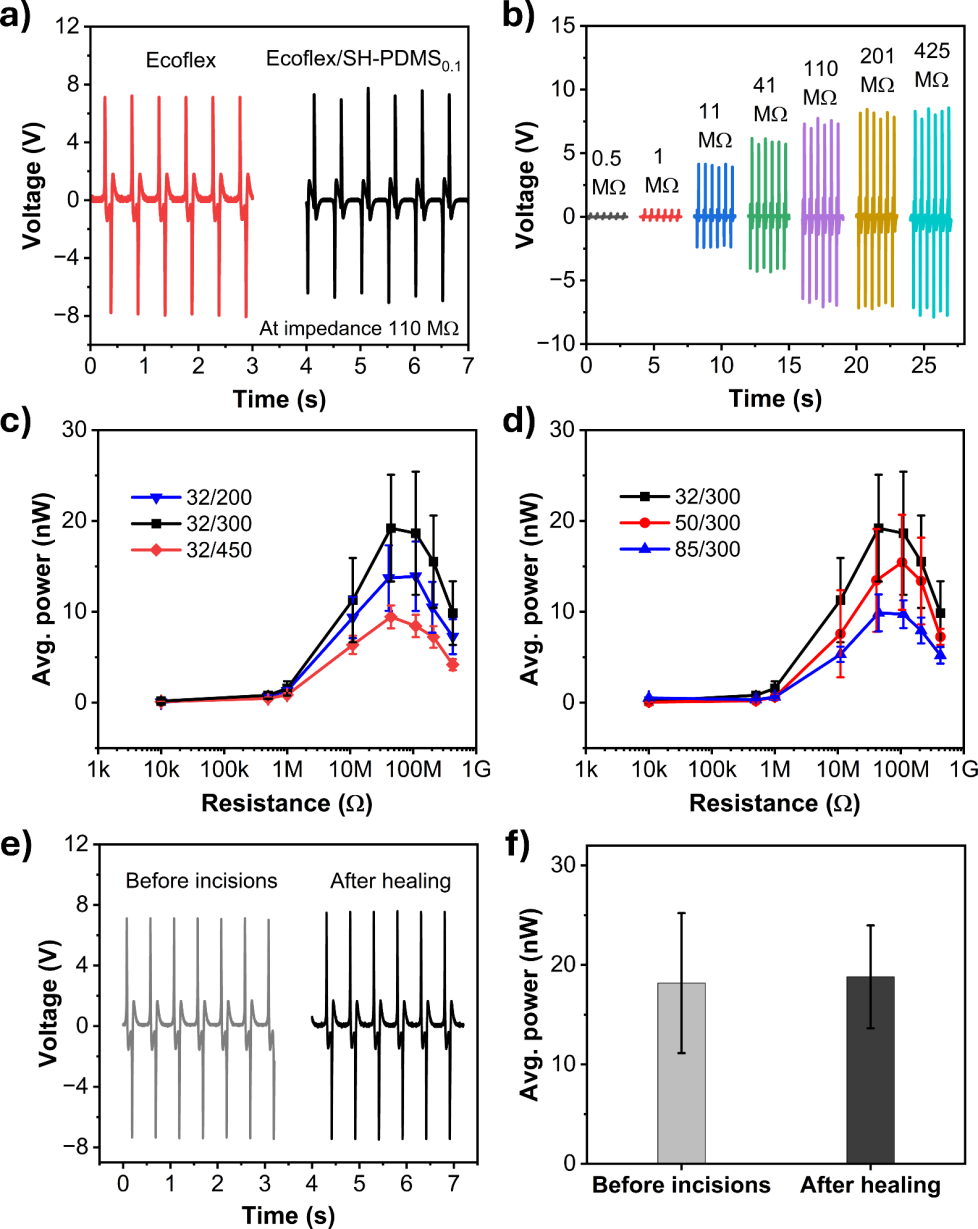
Triboelectric output study. (a) Output voltage of blank Ecoflex (200 μm) and Ecoflex/SH-PDMS_0.1_ (≈32/200 μm) at an external load of 110 MΩ. (b) Output voltage at different external loads of Ecoflex/SH-PDMS_0.1_ (32/200 μm). Variation of output average power connecting with a series of external loads for Ecoflex/SH-PDMS_0.1_ films at different thicknesses of (c) ≈32/200, ≈32/300, and ≈32/450 μm and (d) ≈32/300, ≈50/300, and ≈85/300 μm. Comparison of (e) output voltage and (f) average output power of Ecoflex/SH-PDMS_0.1_ (≈32/300 μm) before incisions and after 24 h of healing at an external load of 104 MΩ.

The output voltage profiles of blank Ecoflex (200 μm) and Ecoflex/SH-PDMS_0.1_ (32/200 μm) films across an external load of 110 MΩ are shown in Figure 6a. Both films show a comparable output voltage, showing an average peak-to-peak voltage (*V*_pk-to-pk_) of ≈15 V. A series of loads is connected to the device to determine the maximum power output through impedance matching. The voltage output at different loads is depicted in Figure 6b. The *V*_pk-to-pk_ sharply increases at a lower load range from 0.5 to 110 MΩ. The *P*_avg_ at various load conditions is calculated using eq 1. It is found that the device shows maximum output power at a load range of 40–110 MΩ (Figure 6c). This load is considered as the impedance-matching optimal load to harvest maximum power from the device. To study the effect of the thickness of the Ecoflex/SH-PDMS_0.1_ triboelectric layer, we explored the TENG output performance by varying the thickness of the SH-PDMS_0.1_ and Ecoflex layers in two different batches. In batch 1, the thickness of the SH-PDMS_0.1_ film layer was 200 ± 4, 300 ± 4, and 450 ± 4 μm, while the Ecoflex layer thickness was kept constant at 32 ± 2 μm for all three samples. In batch 2, the SH-PDMS_0.1_ layer thickness was kept constant at 300 ± 4 μm, and the Ecoflex layer was varied by 32 ± 2, 50 ± 2, and 85 ± 2 μm. We kept a minimum layer thickness of ≈32 μm to obtain a uniform thickness of the Ecoflex layer on SH-PDMS_0.1_. A comparison of the *P*_avg_ values of the devices connecting with variable resistors among different thicknesses of batch 1 and batch 2 is shown in Figure 6c,d. For easy assessment, a comparison table is presented in Table S2, stating the thickness of each layer of Ecoflex and SH-PDMS_0.1_ and the corresponding average output power at an external load of ∼110 MΩ. In batch 1, it was found that mean output power increased with increasing thickness of the SH-PDMS_0.1_ layer from 200 ± 4 to 300 ± 4 μm. However, the output power decreased when the thickness of SH-PDMS_0.1_ was further increased to 450 ± 4 μm. In batch 2, it was observed that the mean outpower decreased with increasing Ecoflex layer thickness from 32 ± 2 to 85 ± 2 μm. The highest TENG output was observed for optimum SH-PDMS_0.1_ and Ecoflex layer thicknesses of 300 ± 4 and 32 ± 2 μm, respectively. It is to be noted that the Ecoflex and PDMS have similar polymer backbones, and thus, the triboelectric output from the integrated thickness of Ecoflex/SH-PDMS_0.1_ layers follows a similar trend. Our results are in good agreement with a previously reported study on the effect of variable thickness of the triboelectric layers on triboelectric output.^61^ The CS-TENG generates a maximum *P*_avg_ of 0.15 ± 0.05 mW m^–2^ across an external load of 110 MΩ.

It was observed that the Ecoflex layer deformed after healing of the bottom PDMS layer. However, it is hard to quantify the TENG performance with the exact deformation level of the Ecoflex layer because the manual incision using a razor blade at different locations is difficult to control and could lead to different widths and lengths of the incision, which causes different deformation levels. Considering the limitation of the deformation analysis, we explored the effect of the deformation on the Ecoflex layer on the triboelectric performance. The Ecoflex/SH-PDMS_0.1_ (32/300 μm) film was incised manually with a razor blade in three different locations approximately 2 mm in length. A schematic diagram of the incision locations (L-1, L-2, and L-3) is shown in Figure S7a). The SEM images of the film surface and cross section of the film after 24 h of healing are presented in Figure S7b,c. After healing, cross-sectional analysis was carried out after completing the TENG output measurement. A DektakXT profilometry 3D map scan was employed to measure the surface roughness at the cutting region before the incision and after 24 h of healing, followed by TENG output measurement. The false-color images of the 3D map scan at the three locations L-1, L-2, and L-3 before the incision and after healing are shown in Figure S7d,e. The *R*_a_ and *R*_q_ values before incisions and after 24 h of healing are included in Figure S7d,e for all three locations. After healing, slight increases in *R*_a_ and *R*_q_ were found in all three locations. The TENG output voltage across a load resistance of 104 MΩ and the *P*_avg_ before incisions and after healing are shown in Figure 6e,f. It was observed that the TENG output performance was not significantly changed due to the deformation of the Ecoflex layer. This can be attributed to the soft and flexible nature of the Ecoflex layer, which can be easily deformed during pressing, causing the top surface to behave the same way as it did before the incisions.

**Figure 7:**
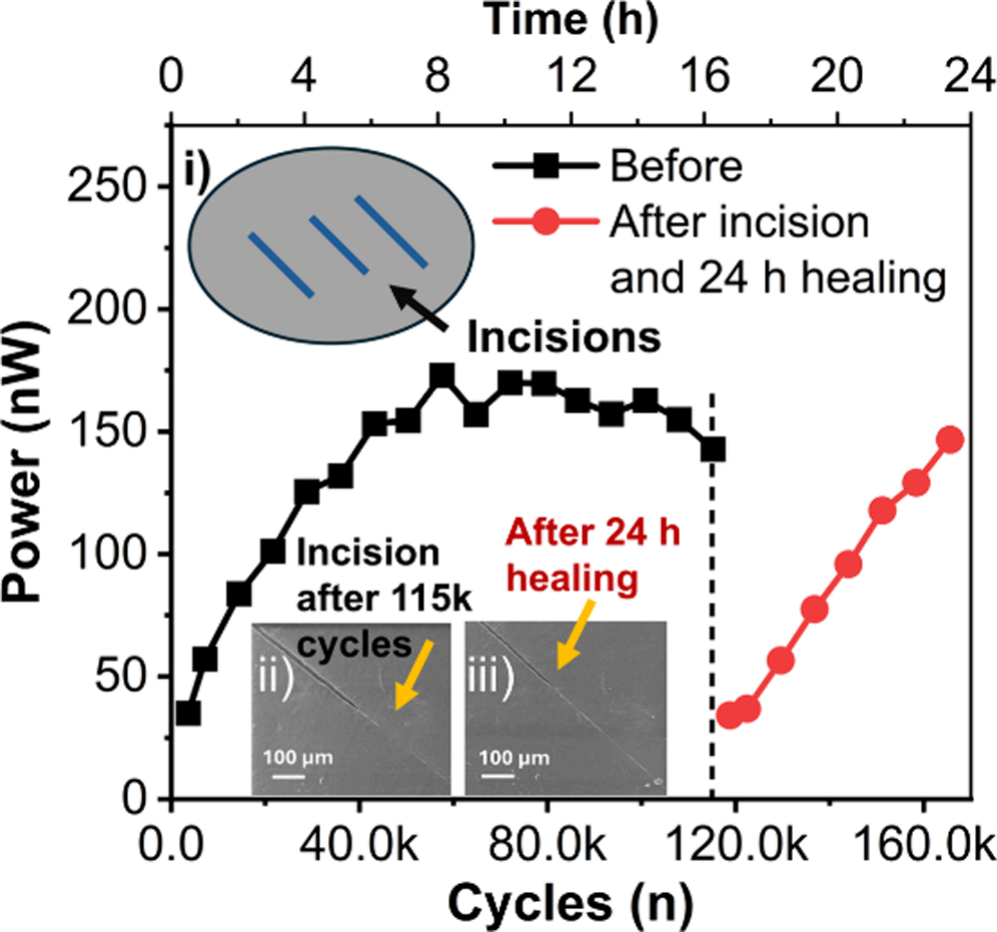
Cyclic stability test for a continuous 16 h, over ≈115 000 cycles, followed by manual incisions using a razor blade at three locations and healing for 24 h and testing again for another 7 h. The first data point for both cases displays the power after 30 min of cycling. Insets: (i) schematic showing three incision locations, (ii) SEM image of an incision, and (iii) healing after 24 h of the Ecoflex/SH-PDMS_0.1_ (≈32/200 μm) film.

To demonstrate the long-term stability of the Ecoflex/PDMS_0.1_ film, a cyclic test was performed continuously for 16 h, over 115 000 cycles. The output power increased slowly upon continuous tapping of Ecoflex/SH-PDMS_0.1_ and nylon 6 layers for 6 h (over 40 000 cycles) and afterward reached a saturation value. This could be due to the fact that the entire surface area of the Ecoflex/SH-PDMS_0.1_ and nylon 6 triboelectric layers had not been fully activated by triboelectrification during the initial tapping cycles. With continuous tapping over long cycles, a greater contact area of the two layers was achieved, leading to a greater amount of triboelectrification and the generation of additional charges. After a long duration of uninterrupted tapping, the film reached a stable state, where electrification happened across the entire active surface of the triboelectric layers, resulting in saturated power output. After 16 h, the Ecoflex/PDMS_0.1_ was removed from the CS-TENG device and manually incised at three locations on the film surface. The SEM image is shown in Figure 7, inset (i), depicting an incised location. The film was kept for 24 h at room temperature and then imaged again to observe the recovery, as depicted in Figure 7, inset (ii). Although the top surface shows a wound mark, the bottom SH-PDMS_0.1_ layer is anticipated to be fully healed, as we observed previously in Figure 3e,h. The healed film was placed in the same CS-TENG device setup as before and tested further for 7 h continually, over 50 000 cycles. The output power was found to be the same as before the incision was made, with close to ≈99% recovery efficiency, before gradually reaching a stable value. Hence, it can be concluded that the healing nature of SH-PDMS_0.1_ in the Ecoflex/PDMS_0.1_ film enabled it to regain its output performance after mechanical damage and provides long-term cyclic stability.

## 4 Conclusions

Self-healable PDMS (SH-PDMS) films can be synthesized by varying the molar ratio of bis(3-aminopropyl)-terminated PDMS (NH_2_-PDMS-NH_2_) and 1,3,5-triformylbenzene (TFB) through the formation of dynamic imine bonds. The SH-PDMS film shows an excellent self-healing capability at ambient temperature. The molar ratio of NH_2_-PDMS-NH_2_ and TFB determines the number of cross-links and optimum healing time. Although the reaction of a higher amount of TFB with NH_2_-PDMS-NH_2_ in SH-PDMS_0.2_ leads to faster healing time, it is challenging to cast multiple films from the same batch, as the cross-linking starts within 2–3 min after mixing. A balance between the ability to cast the film within a reasonable time frame and its ability to heal from mechanical damage is observed for 0.1 mL of TFB (0.8 M) per gram of NH_2_-PDMS-NH_2_ in SH-PDMS_0.1_. However, the inherent stickiness of the self-healable film hinders its application in a contact-separation mode TENG (CS-TENG). A thin coating of Ecoflex elastomer, which belongs to the same family of silicone rubber, not only solves the stickiness issue of SH-PDMS_0.1_ but also retains both the self-healable and triboelectric properties of the material when used as a tribonegative material in a CS-TENG device geometry. The CS-TENG produces a maximum average power density per cycle of 0.15 ± 0.06 mW m^–2^, employing nylon 6 (100 μm) film as the tribopositive material with the Ecoflex/SH-PDMS_0.1_ (≈32/300 μm) film. The Ecoflex/SH-PDMS_0.1_ film shows excellent cyclic stability over 150 000 cycles, and even after recovery from the manual incisions, it was found to regain its original triboelectric output performance. This work leads to the possibility for further modification of the surfaces of self-healable tribonegative polymer films to further improve their triboelectric properties while retaining their self-healing characteristics for robust long-term triboelectric performance.

## Data Availability

Supporting data for this paper is available at the University of Cambridge data repository, Apollo (https://doi.org/10.17863/CAM.112151).

## Supporting Information Available

3D design of mold for SLA 3D printing, schematic of CS-TENG, cross-sectional SEM images of Ecoflex/SH-PDMS_0.1_ films with various thicknesses, XRD patterns of Ecoflex/SH-PDMS_0.1_ film before and after healing and nylon 6 film, DektakXT profilometry 3D map scans of Ecoflex/PDMS_0.1_ and nylon 6, deformation study before incisions and after healing, surface roughness parameters, and TENG output performance of Ecoflex/SH-PDMS_0.1_ films with various thicknesses (PDF)
Video S1: stickiness test of SH-PDMS_0.1_ and Ecoflex/SH-PDMS_0.1_ films (MP4)
NanoDataLyzer is available at: https://doi.org/10.5281/zenodo.13225384

## Abbreviations

TENGs: triboelectric nanogenerators
SH-PDMS: self-healable polydimethylsiloxane
FEP: fluorinated ethylene propylene
PVDF: polyvinylidene fluoride
PTFE: polytetrafluoroethylene
IPDI: isophorone diisocyanate
TPAL: terephthalaldehyde
HDI: hexamethylene diisocyanate
CS: contact-separation
IPA: isopropyl alcohol
DMF: dimethylformamide
TFB: 1,3,5-triformylbenzene
NH_2_-PDMS-NH_2_: bis(3-aminoproyl)-terminated polydimethylsiloxane
Cu: copper
Au: gold
SEM: scanning electron microscopy
SE: secondary electrons
BSE: backscattered electrons
FTIR: Fourier-transform infrared spectroscopy
XRD: X-ray diffraction
SLA: stereolithography
*V*: voltage profile
*P*_avg_: average power output per cycle, *R*, ohmic resistance
*T*: time period per cycle
Δ*t*: total time period for *n* cycles
*R*_a_: arithmetic roughness average
*R*_q_: root-mean-square roughness
