# Supplementary material for: Tailoring of Self-Healable Polydimethylsiloxane Films for Mechanical Energy Harvesting
Source: ACS Appl Energy Mater. 2024 Sep 25;7(19):8185–95. doi: 10.1021/acsaem.4c01275 (PMC11480939; doi:10.1021/acsaem.4c01275)
Supplement: Supplementary file 1 — ae4c01275_si_001.pdf [file ae4c01275_si_001.pdf]

Supporting Information

Tailoring of self-healable polydimethylsiloxane films for mechanical energy harvesting

Kalyan Ghosh[a*], Alexander Morgan[a], Xabier Garcia-Casas[b] and Sohini Kar-Narayan[a*]

[a]*Department of Materials Science & Metallurgy, University of Cambridge, Cambridge CB3 0FS, United Kingdom*

[b]*Nanotechnology on Surfaces and Plasma Group, Materials Science Institute of Seville (CSIC-University of Seville), C/Américo Vespucio 49, 41092, Seville, Spain*

*Corresponding author

Email id: kg533@cam.ac.uk (K. G.); and sk568@cam.ac.uk (S. K.-N.)

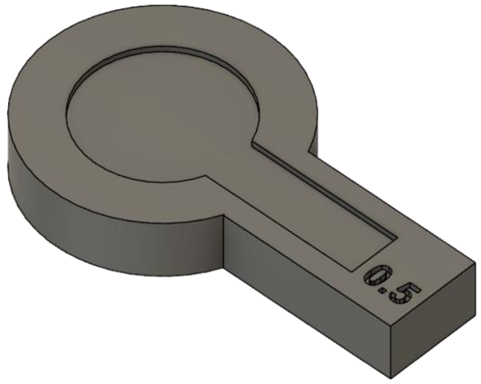

Figure S1. 3D design of a mold for Stereolithography (SLA) 3D printing. The mold with groove thickness of 500 μm, an inner circle diameter of 12.5 mm and a leg length of 13 mm.

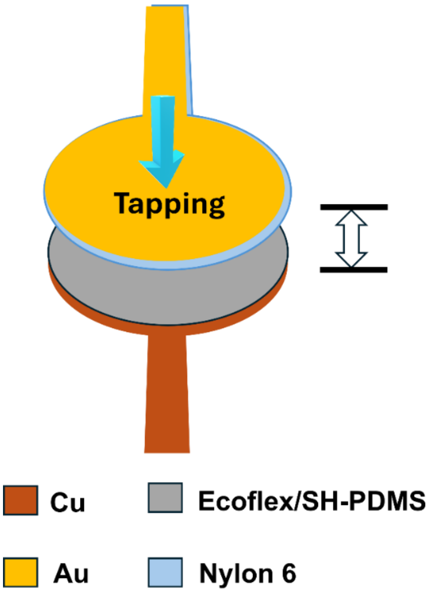

Figure S2. Schematic of the (CS-TENG) device employing Ecoflex/SH-PDMS$_{0.1}$ film as negative triboelectric and nylon 6 as positive triboelectric materials.

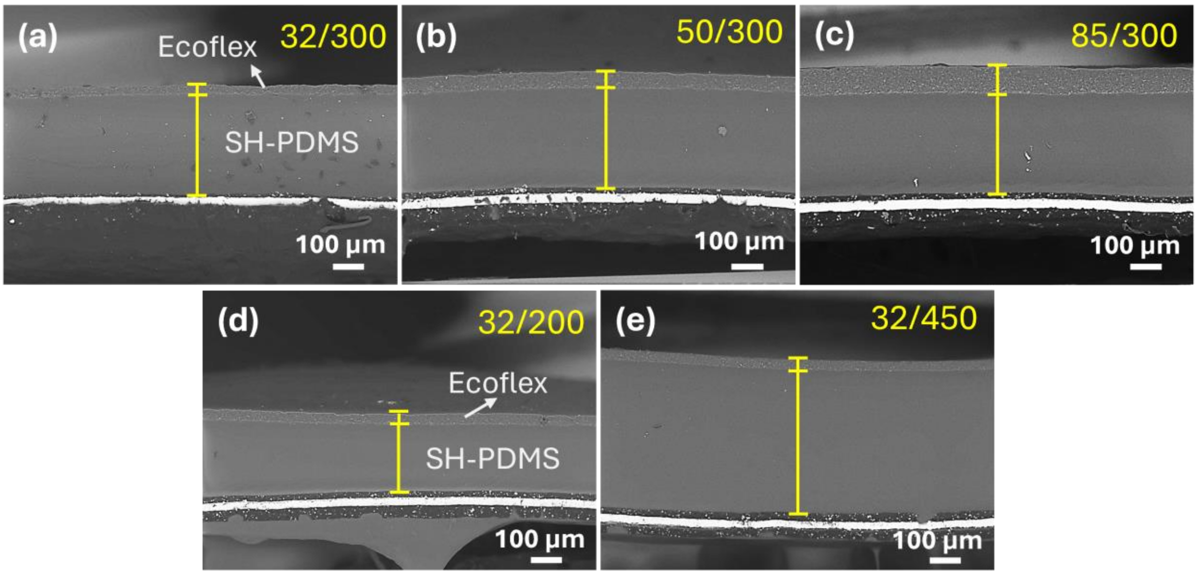

Figures S3. SEM images of the cross-section of SH-PDMS$_{0.1}$ films of the thickness (a) ≈32/300, (b) ≈50/300, (c) ≈85/300, (d) ≈32/200, and (e) ≈32/450 µm.

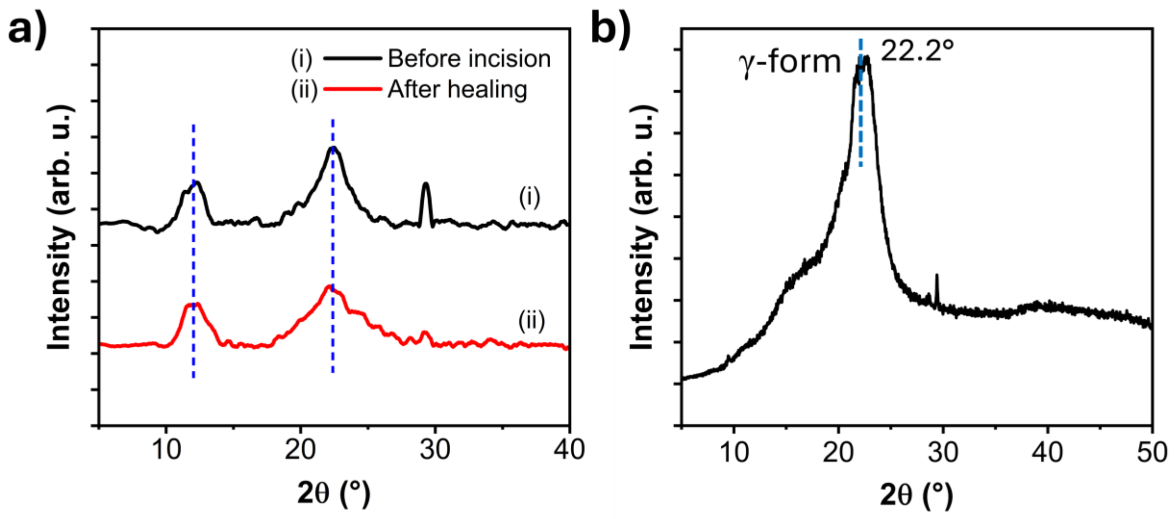

Figure S4. XRD patterns of (a) Ecoflex/SH-PDMS$_{0.1}$ (≈32/300 µm) film before and after healing and (b) nylon 6 film (100 µm).

The XRD pattern of commercial nylon 6 film shows a highly intense broad peak at 2θ = 22.2°, corresponds to the presence of the dominant γ-form crystal in the film. The film does not show any prominent peaks at ≈20 and ≈24° corresponding to the α-form crystal.[1,2] This confirms the commercial film possesses a predominant γ-form which is a metastable phase originating from the hydrogen bonding between parallel chains.[3]

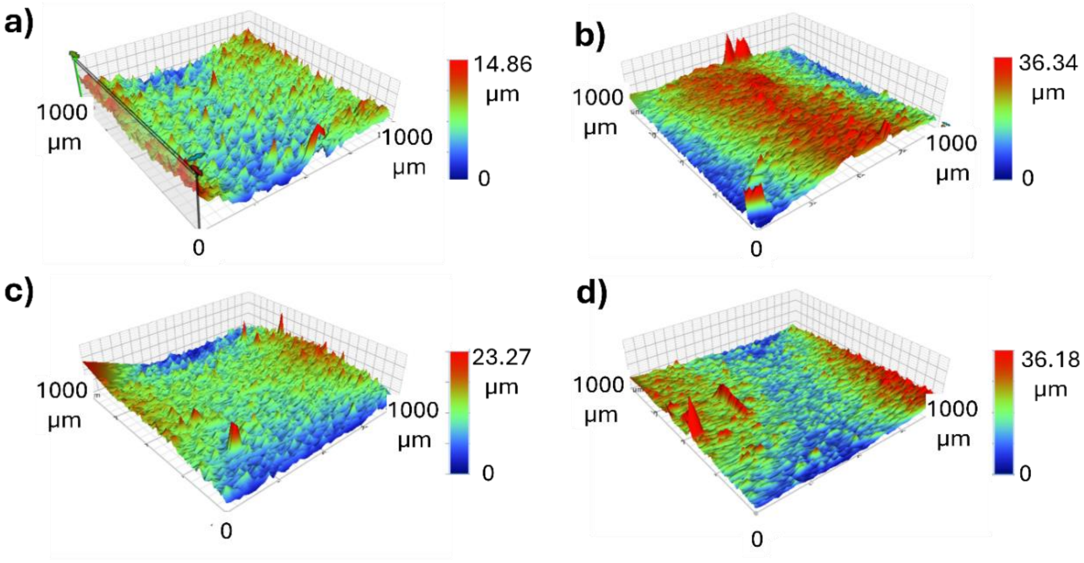

Figure S5. DektakXT profilometry 3D map scan. False-color 3D plots of Ecoflex/PDMS$_{0.1}$ (≈32/300 µm) film of (1 x 1) mm^2 area at four locations (L) (a) L-1, (b) L-2, (c) L-3 and (d) L-4.

The 3D map scan of nylon 6 film (1 × 1 mm^2) in color scale is shown in Figure S6. The surface of the film is found to be very smooth, and the corners are lifted. For the entire scan area, The surface roughness parameters, root mean square (RMS) roughness (R_q) and arithmetic roughness average (R_a) were measured to be 0.2 and 0.2 µm, respectively.

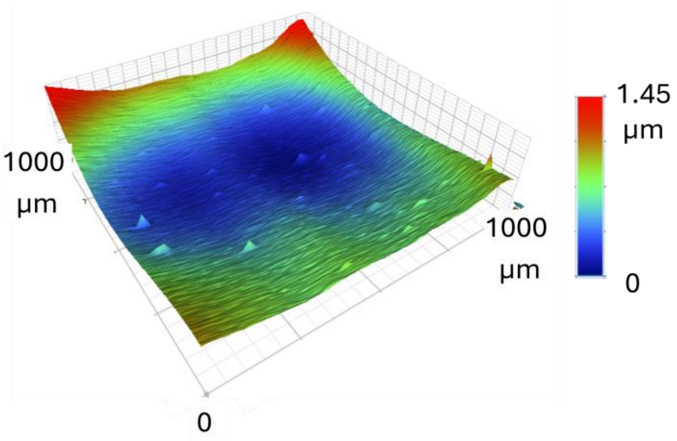

Figure S6. DektakXT profilometry. 3D map scan false-color image of the nylon 6 film (1 x 1) mm^2 area.

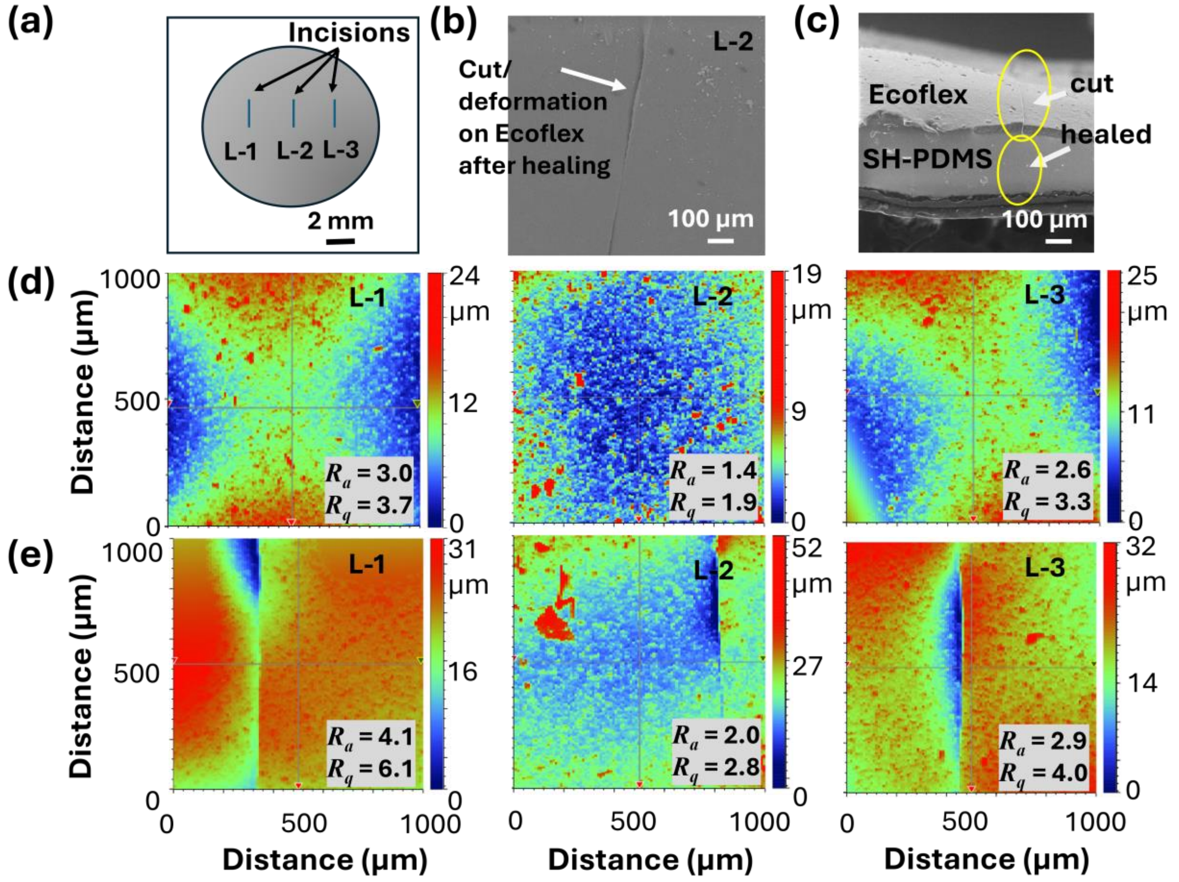

Figure S7. (a) A schematic diagram of the Ecoflex/SH-PDMS$_{0.1}$ (≈32/300 µm) film showing incision locations (L-1, L-2 and L-3), SEM image of the Ecoflex/SH-PDMS$_{0.1}$ film after healing of 24 h at location L-2, (b) top surface and (c) cross-section. DektakXT profilometry 3D map scan false-color images at three locations L-1, L-2 and L-3 (d) before incisions and (e) after 24 h of healing.

Table S1. Surface roughness parameters at four locations, and their average and standard deviation**.**

	Root mean square roughness, R_q (µm)	Arithmetic roughness average, R_a (µm)
L-1	1.8	1.4
L-2	3.3	2.7
L-3	2.4	1.9
L-4	2.2	1.7
Avg.	2.425	1.925
Std.	0.634	0.556

Table S2. Comparative study of TENG output performance of Ecoflex/SH-$PDMS_{0.1}$ films with various thicknesses.

Batches	Thickness (µm)			Mean Power (nW), at 110 MΩ
	Ecoflex	SH-$PDMS_{0.1}$	Total	
Batch-1	32 ± 2	200 ± 4	233 ± 6	13.9 ± 3.8
	32 ± 2	300 ± 4	333 ± 6	18.6 ± 6.7
	32 ± 2	450 ± 4	483 ± 6	8.4 ± 1.2
Batch-2	32 ± 2	300 ± 4	333 ± 6	18.6 ± 6.7
	50 ± 2	300 ± 4	345 ± 6	15.4 ± 5.2
	85 ± 2	300 ± 4	385 ± 6	9.7 ± 1.5

References

(1) Jose, M. V; Steinert, B. W.; Thomas, V.; Dean, D. R.; Abdalla, M. A.; Price, G.; Janowski, G. M. Morphology and Mechanical Properties of Nylon 6/MWNT Nanofibers. *Polymer* **2007**, *48* (4), 1096–1104.

(2) Kimura, N.; Kim, B.-S.; Kim, I.-S. Effects of Fe^{2+} Ions on Morphologies, Microstructures and Mechanical Properties of Electrospun Nylon-6 Nanofibers. *Polym. Int.* **2014**, *63* (2), 266–272.

(3) Murase, S.; Matsuda, T.; Hirami, M. Intrinsic Birefringence of γ-Form Crystal of Nylon 6: Application to Orientation Development in High-Speed Spun Fibers of Nylon 6. *Macromol. Mater. Eng.* **2001**, *286* (1), 48–51.
